# In cryptozoospermia or severe oligozoospermia is sperm freezing useful?

**DOI:** 10.1186/2051-4190-24-15

**Published:** 2014-10-02

**Authors:** Julien Bessonnat, Sophie Brouillet, Sarah Sintzel, Pierre Gillois, Ulrike Bergues, Caroline Boutte-Busquet, Claire Thomas-Cadi, Sylviane Hennebicq

**Affiliations:** Laboratoire d’Aide à la procréation-CECOS, University Hospital of Grenoble, Grenoble, France; University Joseph Fournier, Grenoble, France; Andrology, Genetic and Cancer Team, AGIM-FRE 3405, Faculty of Medicine, Grenoble, France; UMR 5525, Technics of Medical Engineering and Complexity, Grenoble, France

**Keywords:** Cryptozoospermia, ICSI, Male infertility, Oligozoospermia, Sperm bank, Cryptozoospermie, ICSI, Infertilité masculine, Oligozoospermie, Congélation spermatique

## Abstract

**Background:**

Intracytoplasmic Sperm Injection (ICSI) is an Assisted Reproduction Technique (ART) which offers the chance to conceive to patients presenting very low sperm counts (cryptozoospermia/severe oligozoospermia). Sperm freezing before the oocyte pick-up, can prevent from a lack of spermatozoa on the day of the ICSI. It can avoid the cancellation of the ICSI or the use of TESE (Testicular sperm extraction). The objective of this study was to analyse the practice of sperm freezing for these patients in our center over 8 years and the rate of use of these frozen sperms. We also compared the outcome of ICSIs with frozen versus ejaculated sperm.

**Material and methods:**

We performed a retrospective epidemiological study between 2004 and 2011. We recruited all the patients having a sperm count below 1 Million/mL and who were waiting for their first ICSI attempt.

**Results:**

169 patients were recruited: 84 cryopreserved their sperm before the ICSI (secured ICSI) while 85 did not (non-secured ICSI). Both groups were split in cryptozoospermia (<10^3^ spermatozoa/ml): 19 and 17 patients respectively, very severe oligozoospermia (10^3^–10^5^/ml): 37 and 13 patients, and severe oligozoospermia (10^5^–10^6^/ml): 28 and 55 patients. The part of secured ICSI significantly increased from 29% during 2004–2007 to 74% during 2008–2011(p = 0.0029) and the frozen sperm was used in 5.9% of the cases. Median age was significantly higher in the non secured ICSI group (33.57 vs 35.52 for men, p = 0.0069 and 30.45 vs 32.26 for women, p = 0.025) but no significant difference was found in the outcome of the ICSI between frozen-thawed sperm and fresh ejaculated sperm.

**Conclusion:**

Sperm freezing before ICSI for severe oligozoospermic and cryptozoospermic patients significantly increased in our practice but the rate of use remain very low. This encourages to define more accurate criteria leading to sperm freezing.

**Electronic supplementary material:**

The online version of this article (doi:10.1186/2051-4190-24-15) contains supplementary material, which is available to authorized users.

## Resume

**Contexte:**

L’ICSI (Intracytoplasmic Sperm Injection) permet aux patients porteurs d’une oligozoospermie sévère ou d’une cryptozoospermie d’espérer concevoir un enfant. Ces patients courent parfois le risque de survenue d’une azoospermie constatée le jour de l’ICSI. L’organisation préalable d’une ou plusieurs congélations spermatiques peut permettre de prévenir ce risque. L’objectif de cette étude était d’évaluer cette pratique de congélation spermatique de sécurité sur une période prolongée dans notre centre et d’évaluer le taux d’utilisation de ces spermes congelés et les chances de grossesse au cours des ICSI, qu’elles soient faites avec les spermatozoïdes frais ou congelés et selon la gravité de l’oligozoospermie.

**Materiel et methodes:**

Il s’agit d’une étude rétrospective monocentrique descriptive de 2004 à 2011. Les critères d’inclusion étaient une numération spermatique inférieure à 1 Million/mL pour une première tentative d’ICSI.

**Resultats:**

169 patients ont été recrutés: 84 ont bénéficié de congélation de spermatozoïdes avant l’ICSI (ICSI sécurisée) et 85 patients n’en ont pas bénéficié (ICSI non sécurisée). Au sein de ces deux groupes, les patients ont été répartis en 3 sous-groupes en fonction de leur concentration spermatique: cryptozoospermie (<10^3^ spermatozoïdes/ml), 19 et 17 patients, oligozoospermie très sévère (10^3^–10^5^/ml), 37 et 13 patients, et oligozoospermie sévère (10^5^–10^6^/ml): 28 et 55 patients. Durant la période analysée nous avons observé une augmentation significative du nombre de congélations spermatiques préalables à une ICSI, passant de 29% des tentatives pour 2004–2007 à 74% des tentatives pour 2008–2011 (p = 0.0029. Seulement 5,9% des patients ont utilisé les spermatozoïdes congelés. L’âge moyen des patients s’est avéré significativement plus élevé dans le groupe des ICSI sans congélation spermatique que avec congélation spermatique (33.57 vs 35.52 chez les hommes, p = 0.0069 et 30.45 vs 32.26 chez les femmes, p = 0.025) sans qu’il y ait de différence significative dans l’issue des tentatives d’ICSI.

**Conclusion:**

La congélation spermatique avant ICSI dans le cas des oligozoospermies sévères et cryptozoospermies est devenue presque systématique dans notre pratique. Pour autant l’utilisation de ces spermatozoïdes congelés demeure très peu fréquente. Ceci nous engage à poursuivre l’étude de ces situations afin de définir des critères cliniques et/ou biologiques justifiant ces congélations souvent itératives.

## Background

Since the 90’s the Intracytoplasmic Sperm Injection (ICSI) has considerably improved the Assisted Reproductive Technologies [1]. This technique allows men with a very low sperm count to achieve their project of parenthood. Cryptozoospermic patients and non-obstructive azoospermic patients can successfully undergo this technique because even these patients can produce extremely rare sperms [2] leading to the notion of virtual azoospermia [3]. In these cases, only very meticulous methods of sperm searching in repeated semen samples allow to retrieve these rare sperms [4–6].

ICSI remains difficult in this population of patients, because we can face an absence or an insufficient number of spermatozoa in the semen on the day of the oocyte pick-up. The ICSI may thus be cancelled. Several centers have organized an alternative TESE (Testicular or Epididymal Sperm Extraction) synchronous to oocyte pick-up to prevent cancelling the ICSI in these cases. Nowadays another option through oocyte vitrification for further use (with a planified TESE or frozen ejaculated sperm) may also be offered but it only postpones the resolution of the lack of sperm.

In other centers, synchronous ICSI-TESE is not possible and they have developed a politic of extended search in repeated ejaculates according to Koscinski *et al.* [7] combined to microfreezing before the ovarian stimulation has started. This option of “security straw” freezing avoids not only cancellation of the ICSI but also a premature TESE [7, 8]. Extended to the patients who have very low sperm count, this micro-freezing of security straws can also prevent further azoospermia of these patients. But is this practice really useful for the patients and can it be managed over long periods?

In order to evaluate this practice, we designed a retrospective study to address the following objectives:

- The main objective was to describe the evolution of sperm banking over a long period and analyze the rate of use of the banked sperm in the specific group of patients carrying cryptozoospermia or severe oligozoospermia
- The secondary objective was to compare the success rates of the ICSIs done with fresh vs frozen sperm when very few motile sperm is available.

## Material and methods

### Patients

We conducted a retrospective epidemiological study in the IVF and CECOS center of Grenoble University Hospital. Patients who had a sperm count of 1 × 10^6^/mL or less and performed their first ICSI attempt between January 2004 and December 2011 were included in the study. The patients who had a sperm storage due to gonadotoxic treatment were excluded from the study. We restricted the study to the first ICSI attempts in order not to introduce a bias of recruitment in the ICSI success rate analysis. The study was ethically ruled through a non-opposition procedure, since it did not contain any extra sampling procedure or specific identified risk for the patients.

The data were extracted from a home-made Access® IVF database for the years 2004 to 2009 and from the Medifirst® system for the years 2009 to 2011. We also consulted the paper records of our sperm bank to ensure that we included all the patients who fitted the inclusion criteria. Once the data had been extracted from the paper or electronic files, we divided our data in two groups of patients: patients having or not sperm storage in the months preceding the first ICSI (respectively named secured ICSI and non secured ICSI groups). For each man, we collected, the date of birth, the date of the ICSI and the sperm count at the day of the ICSI. The patients were split in three groups according to the severity of the oligozoospermia: cryptozoospermia (spermatozoa observed only in the pellet with sperm concentration always <0.001 × 10^6^/mL), very severe oligozoospermia (sperms observed sparsely in a few fields at × 400 magnification of the fresh preparation with sperm concentration from 0.001 to 0.1 × 10^6^ spermatozoa/mL) and severe oligozoospermia (sperms observed sparsely in all the fields at × 400 magnification in the fresh preparation with sperm concentration from 0.1 to 1 × 10^6^ spermatozoa/mL).

For all the ICSIs, we collected:

- the date of birth of the female partner and the date of oocyte pick-up,
- the number of oocytes retrieved, metaphase two oocytes injected and two-pronuclei zygotes obtained (to determine the fertilization rate),
- the number of cleaved embryos on day 2 (to determine the cleavage rate),
- the number of transferred or frozen embryos,
- the number of ongoing clinical pregnancy (developing embryo with cardiac activity at the echography 1 month after the ICSI).

In the secured ICSI group, the date of the sperm storage and the number of straws stored and/or thawed were also compiled.

### Sperm analysis and freezing procedure

Sperm analysis was performed according to the WHO recommendations: WHO 1999 [9]. Before freezing, the semen was diluted (v/v: 1/0.7) at room temperature with SpermFreeze® (FertiPro, Beernem, Belgium) with mild and continuous shaking. In order to increase the sperm concentration, the sample was frequently centrifuged (10 min at 200G) before adding the freezing medium. The mix was then loaded in 0.3 mL straws. Samples were cooled from room temperature to -150°C following an automated (Planer Kryo 560®, CryoBioSystem) 3 slopes cooling procedure (from room temperature to -8°C at -5°C/min, from -8°C to -25°C at -10°C/min and from -25°C to -150°C at -20°C/min). When -150°C was reached, the straws were immediately plunged and stored in liquid nitrogen.

### In vitro fertilization by ICSI

The ovarian stimulations, the oocyte retrieval and the ICSI procedure were conducted according to the standardized protocols as previously described [10, 11]. The semen was collected by masturbation on the day of the ICSI and prepared using discontinuous three layers density gradient (PureSperm 100® Nidacon/Sperm Preparation Medium® Origio, v/v): 1 mL 1/0, 1 mL 0.7/0.3 and 1.5 mL 0.4/0.6). In case of cryptozoospermia or a sperm motility below 20%, the layers were 0.4 mL of 1/0, 0.4 mL 0.7/0.3 and 0.6 mL of 0.4/0.6. If no spermatozoon was observed in the first 10 μL of the ejaculate, a two layers density gradient centrifugation (0.4 mL of 1/0 and 0.6 mL of 0.4/0.6) or a simple centrifugation (10 min, 200G) was realized and, if there still was no spermatozoon observed, a second retrieval was proposed. After two unsuccessful samples, one or more straws were thawed.

Oocytes were retrieved in Flushing Medium® (Origio) at day 0 and moved in IVF® Medium (Origio). Then, the cumulus-corona cells were removed by exposure to hyaluronidase (JCD® 80UI/mL) diluted (v/v 1/1) with IVF® medium (Origio). Each metaphase II oocyte was disposed in a drop of Sperm Preparation Medium® (Origio), the prepared sperm were put in a drop of PVP® (Origio) and the ICSI was performed under inverted microscopic control (Axiovert 35 M®, Zeiss or IX81® Olympus). After the injection, the oocytes were placed in ISM1® medium (Origio) and incubated at 37°C (5% CO_2_, cytoperm 2®, Thermo Scientific Heraeus).

Appearance of 2 pronuclei was noted at day 1 and the embryo cleavage was quoted at day 2 and further. The embryos were transferred between day 2 and day 5. If possible, embryos were moved in BlastAssist® (Origio) at day 3 to achieve blastocyst stage. Supernumerary embryos were frozen either at day 2, 3 or 5.

### Statistical analysis

R 3.0.2 or Excel 2013 software was used for statistical analysis. χ2 test was used for qualitative variables and Student’s test was applied for quantitative variables with a significance level at 5% for both tests.

## Results

### Description of the analyzed population

169 couples who had their first ICSI attempt between 2004 and 2011, with a sperm count of one million/mL or less were included in the study. ICSI without any sperm freezing (non secured ICSI) was performed for 84 couples, while the other 85 men had at least one sperm sample frozen before the first ICSI attempt (secured ICSI). The general and seminal characteristics of each group are described in Table  1. The men who cryopreserved their sperm before ICSI were significantly younger (mean age was 33.57 in secured ICSI group vs 35.52 for the non secured ICSI group, p < 0.05). The partners in the first group were also younger with approximately the same difference in age (30.45 years vs 32.26, p < 0.01).

**Table 1:** **General characteristics and sperm parameters of the analyzed population**

	Secured ICSI group (n = 84)			Non secured ICSI group (n = 85)			p-value
	Mean	Median	min-max	Mean	Median	min-max	
Female age (year)	30.45	30.50	21–40	32.26	32.00	22–42	0.0069**
Male age (year)	33.57	33,00	23–56	35.52	35.00	22–49	0.0254*
Sperm count (×10^6^/mL)	0.192	0.006	0–1	0.399	0.200	0–1	0.000263***
Cryptozoospermia number of patients	19/84 (22.6%)			17/85 (20.0%)			0.819
Sperm count (×10^6^/mL)	0.0003	0.0002	0–0.0009	0.0002	0.0001	0–0.0009	0.187
Very Severe oligozoospermia number of patients	37/84 (44.0%)			13/85 (15.3%)			0.0000863***
Sperm count (×10^6^/mL)	0.0095	0.0040	0.001–0.006	0.0030	0.0020	0.001–0.007	0.0177*
Severe oligozoospermia number of patients	28/84 (33.3%)			55/85 (64.7%)			0.0000867***
Sperm count (×10^6^/mL)	0.564	0.600	0.1–1	0.616	0.800	0.1–1	0.494

Secured ICSI: ICSI with previously frozen sperm; non secured ICSI: ICSI without previously frozen sperm.

*Significant p values at 5%, **Significant p value at 1%, ***Significant p value at 0.1%.

Mean sperm count in the secured ICSI group was significantly lower than in the non secured ICSI group (0.192 × 10^6^/mL versus 0.399 × 10^6^/mL, p < 0.001). The part of the cryptozoospermic patients was not different among both groups (22.6% vs 20.0%, p = NS); while the secured ICSI group contained mainly very severe oligozoospermia (44.0% vs 15.3%, p < 0.001) and the non secured ICSI group mainly non severe oligozoospermia (64.7% vs 33.3, p < 0.001). Mean sperm count for severe oligozoospermia or cryptozoospermia was not different in both groups (0.564 × 10^6^/mL vs 0.616 × 10^6^/mL, p = NS for severe oligozoospermia and 0.0003 × 10^6^/mL vs 0.0002 × 10^6^/mL, p = NS for cryptozoospermia), while the mean sperm count among very severe oligozoospermia was significantly lower in the non secured-ICSI group than in the secured-ICSI group (0.0030 × 10^6^/mL vs 0.0095 × 10^6^/mL, p < 0.05).

### The practice of sperm banking over the analyzed period

**Figure 1:**
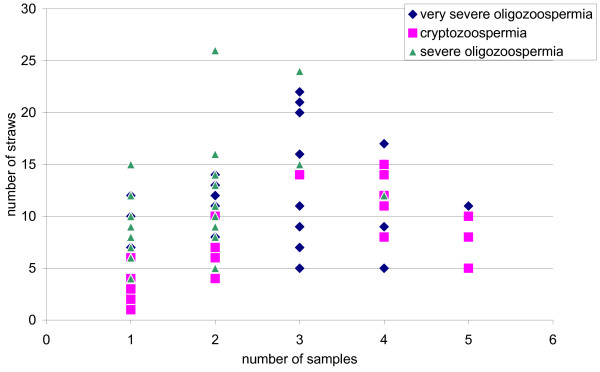
**Distribution of the number of straws according to the number of frozen samples in the three subgroups of patients.**

We secondly analyzed the data of sperm freezing and banking over the period (Table 2). Considering the whole population, an average of 2.23 sperm samples was frozen for each patient. The mean number of straws was of 10.04. When analyzing these data among the different subgroups, we observed that the mean number of samples was lower in case of severe oligozoospermia, while the lowest number of straws was observed in the cryptozoospermia group. The mean number of samples was not very different when considering each subgroup of patients (respectively 2.73 for cryptozoospermia; 2.46 in case of very severe oligozoospermia and 1.57 for severe oligozoospermia) but 75% of the severe oligozoospermic patients performed only one sample, while 75% of the cryptozoospermic ones came two to 5 times in the lab for sperm freezing. We also noticed that the patients with very severe or severe oligozoospermia had at least 4 straws in our tanks while several of the cryptozoospermic patients had only one straw. Analysis of the distribution of the number of straw according to the number of frozen samples among the different subgroups (Figure 1) revealed that the patients having the lowest amount of frozen straws were those for whom only one sample could be frozen, often despite several venues in the lab.

**Figure 2:**
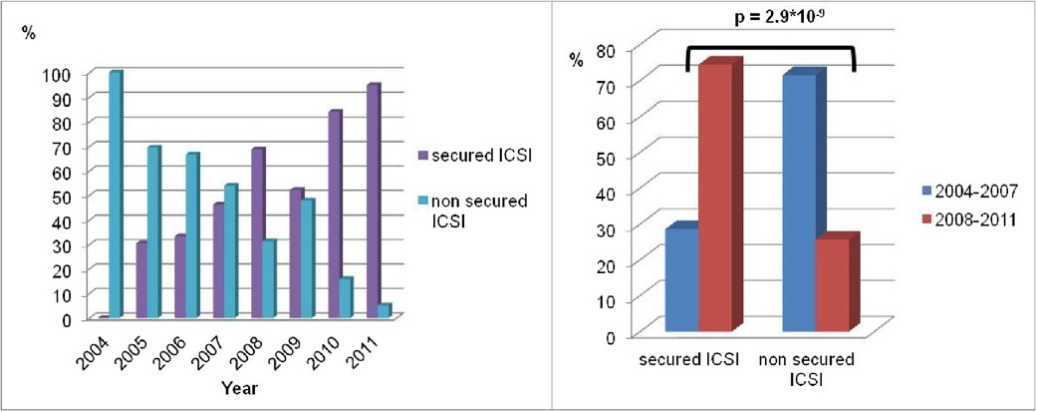
**Evolution of the distribution of secured and non-secured ICSIs between 2004 and 2011.** Left: Evolution between 2004 and 2011. Right: Comparison between the period 2004–2007 and 2008–2011.

We looked further at the distribution of secured and non secured ICSIs along the time between 2004 and 2011 (Figure 2). The global analysis over the whole period clearly showed an increase of sperm freezing in case of severe oligozoospermia as illustrated by the increase of secured ICSI over the time (Figure 2 left). In 2004, none of the patient had his sperm frozen before an ICSI. Between 2005 and 2010, the rate of secured ICSIs for oligozoospermia raised from 30% to 95% and in 2011, only one oligozoospermic man underwent an ICSI in our center without any previous frozen sperm. We divided the 8 years period in two equal periods of 4 years and observed a significant difference in the percentage of secured ICSIs between the two periods 2004–2007 and 2008–2011 (29% vs 74% of secured ICSI for oligozoospermia, p < 0.001, Figure 2 right).

**Table 2:** **Characteristics of sperm cryopreservations**

Colonne1	Mean	min-max	1st quartile	median	3rd quartile	Patients
Frozen samples per patient	2.23	1–5	1	2	3	All the patients with sperm storage (n = 84)
	2.73	1–5	1	2	4	Cryptozoospermic patients (n = 19)
	2.46	1–5	2	2	3	Very severe oligozoospermic patients (n = 37)
	1.57	1–4	1	1	2	Severe oligozoospermic patients (n = 28)
No of straws per patient	10.04	1–26	7	10	12,25	All the patients with sperm storage (n = 84)
	7.53	1–15	4	7	10.5	Cryptozoospermic patients (n = 19)
	10.92	4–22	8	10	13	Very severe oligozoospermic patients (n = 37)
	10.57	4–26	7.75	9.5	12.25	Severe oligozoospermic patients (n = 28)

### Rate of use of the banked sperm

Among the 84 couple who had frozen sperm before the ICSI, thawing sperm was necessary for only 5 patients, which corresponded to 5.9% of use of the frozen sperm (Table 3). The reasons leading to thaw straws were: absence or insufficient number of spermatozoa in the fresh ejaculate (after two sperm sampling) on the day of the ICSI attempt (4 patients) or remaining bacteria in sperm before the attempt, despite repeated and adapted antibiotics (1 patient). Sperm samples were thawed among the three categories of oligozoospermic patients but the highest rate of use was observed among the cryptozoospermic groups (10.5%).

**Table 3:** **Rate of use of the frozen sperm among the secured ICSI population**

	Number of patients whose frozen sperm was thawed to perform the ICSI (rate of use)
All the patients	5/84 (5.9%)
Cryptozoospermia	2/19 (10.5%)
Very severe oligozoospermia	1/37 (2.7%)
Severe oligozoospermia	2/28 (7.1%)

### Do the patients have the same chances of pregnancy as the sperm parameters decrease?

We compared the ICSI attempts in the three groups of men (Table 4) and found a significantly higher embryo cleavage rate in case of severe oligozoospermia compared to very severe oligozoospermia or cryptozoospermia (76% vs 64%, p = 0.02 and 76% vs 66%, p = 0.02 respectively). We also noticed a significantly higher number of frozen embryos in severe oligozoospermia compared to very severe oligozoospermia (2.12 versus 0.86, p = 0.00031). But analysing the implantation rates revealed no significant difference among the groups, neither was observed a difference in fertilization rate, number of embryo transferred, pregnancy rate or birth rate per ICSI cycle.

**Table 4:** **ICSI outcome according to the severity of the oligozoospermia**

	Cryptozoospermia (group 1) n = 36		Very severe oligozoospermia (group 2) n = 50		Severe oligozoospermia (group 3) n = 83		P value (group 1 vs 2)	P value (group 1 vs 3)	P value (group 2 vs 3)
	Mean (total)	IC 95%	Mean (total)	IC 95%	Mean (total)	IC 95%			
Women’s age (years)	31.7	± 1.51	30.8	± 1.13	31.6	± 0.96	0.4	0.89	0.30
Men’s age (years)	34.0	± 1.75	34.7	± 1.71	34.7	± 1.19	0.59	0.54	0.99
Sperm count (10^6^/mL)	0.00028	± 0.000092	0.0078	± 0.0037	0.60	± 0.070	0.00026*	2.5 × 10 ^-28^*	5.2*10 ^-28^*
Use of thawed sperm	2/36 (5.5%)		1/50 (2.0%)		2/83 (2.4%)		0.57	0.58	1
No of oocytes retrieved	10.17 (366)	± 1.95	11.30 (554)	± 2.33	10.51 (862)	± 1.27	0.47	0.77	0.56
No of oocytes injected	8.17 (294)	± 1.85	9.14 (448)	± 1.44	8.71 (723)	± 1.07	0.42	0.62	0.64
Fertilization rate	0.56 (165)	± 0.07	0.56 (236)	± 0.07	0.64 (455)	± 0.05	0.94	0.10	0.08
Cleavage rate	0.64 (200)	± 0.08	0.66 (283)	± 0.06	0.76 (541)	± 0.04	0.71	0.02*	0.02*
No of transferred embryos	1.31 (47)	± 0.22	1.40 (70)	± 0.16	1.37 (114)	± 0.14	0.49	0.61	0.80
No of frozen embryos	1.33 (48)	± 0.70	0.86 (43)	± 0.34	2.12 (176)	± 0.57	0.24	0.09	0.00031*
Pregnancy rate per ICSI cycle	0.19 (7)	± 0.13	0.32 (16)	± 0.13	0.23 (19)	± 0.09	0.19	0.68	0.25
Implantation rate	0.20	± 0.14	0.24	± 0.03	0.19	± 0.09	0.68	0.90	0.49
Birth rate per ICSI cycle	0.14 (5)	± 0.11	0.18 (9)	± 0.11	0.23 (19)	± 0.09	0.61	0.26	0.50

*Significant p values below 5%. (Student’s *t* test).

### Success rate of the ICSIs using fresh or frozen sperm

We compared the results of ICSI (Table 5) with fresh ejaculated sperm (group A) or thawed sperm (group B). Since there was no difference between secured and non secured ICSI with fresh ejaculated sperm (data not shown), the results of these two groups were pooled in group A. The analysis revealed no difference for all the data analyzed (age of the patients, number of oocytes collected or injected, fertilization rate, and cleavage rate, number of transferred or frozen embryos, pregnancy rate, implantation rate and birth rate).

**Table 5:** **Analysis of the ICSIs with ejaculated sperm or thawed sperm**

	ICSIs with fresh sperm (group A)		ICSIs with thawed sperm (group B)		p-value
	Mean (total)	IC 95%	Mean (total)	IC 95%	
Women’s age (years)	31.4	±0.68	29.8	±1.80	0.159
Men’age (years)	34.6	±0.88	33.4	±3.75	0.576
Sperm count (/ml)	298 × 10^3^	±58 × 10^3^	242 × 10^3^	±280 × 10^3^	0.717
No of oocytes retrieved	10.68 (1741)	±1.03	10.25 (52)	±6.73	0.918
No of oocytes injected	8.70 (1418)	±0.79	9.4 (47)	±5.7	0.823
Fertilization rate	0.60 (833)	±0.04	0.57 (23)	±0.13	0.715
Cleavage rate	0.70 (995)	±0.04	0.77 (29)	±0.23	0.628
No of embryos transferred	1.37 (225)	±0.10	1.20 (6)	±0.45	0.446
Number of frozen embryos	1.57 (257)	±0.35	2.00 (10)	±1.75	0.658
Pregnancy rate per ICSI cycle	0.25 (41)	±0.07	0.20 (1)	±0.39	0.642
Implantation rate	0.21	0.06	0.20	±0.35	0.962
Birth rate per ICSI cycle	0.20 (32)	±0.06	0.20 (1)	±0.35	0.862

No significant difference for all parameters. (Student’s *t* test).

## Discussion

### What about routine sperm storage in case of oligozoospermia?

Routine use of sperm storage started in the 60’s for patients who undergo gonadotoxic treatment [12, 13]. More recently, sperm storage has also been proposed to patients presenting a very low sperm count or suspected at high risk of rapid decrease in the sperm count (possibly linked to several genetic origins of the oligozoospermia) and asking for Assisted Reproductive Techniques [13]. This practice was also reinforced in our center by several cases of severe male infertility which led to azoospermia even before the first ICSI had been done. Nevertheless, it is a time consuming practice and only few studies were available so far to evaluate the real benefit for the patients. The first important one was by Lahav-Baratz et al and described the interest of sperm pooling and cryopreservation before the ICSI [8].

When comparing our sperm storage data to the literature, we see that the amount of samples per patient is not different from those observed in case of sperm storage before gonadotoxic treatment [14–16], but the number of straws is lower. In case of cancer, most of the patients show normal or subnormal sperm count. It is thus not necessary to concentrate the sperm cells before freezing, this allows to make more straws than in the case of severe oligozoospermia or cryptozoospermia. The analysis of the evolution of the sperm storage during ART in our center shows a rising proposal of cryopreservation before ICSI for severe oligozoospermia. Between 2004 and 2011, the percentage of patients who were offered the opportunity of sperm storage, to prevent future azoospermia during the ICSI protocols, increased from 30% to 95%. A significant difference of age is observed among our patients according to sperm banking before ICSI or not. The first group is 1.5 year younger for men and 2 years younger for their female partner. It is surprising because we may be more prone to propose sperm banking to older men to prevent further decrease, while our analysis reveals the opposite. A possible explanation may be that the high degree of oligozoospermia leads to a faster ICSI proposal to these couples. Ping and collaborators analyzed the practice of sperm storage and reuse in China between 2003 and 2008 [17]. The main part of their indication for sperm banking was the risk of sperm collection failure or couple separation on the day of the ICSI, while oligozoospermia represented 4.8% of ART patients with sperm banking (66/1374). The mean sperm count for their oligozoospermic patients was 3.1 × 10^6^.

It is noticeable that we observed a significantly higher proportion of very severe oligozoospermic patients and a lower rate of severe oligozoospermic patients in the secured ICSI group. It is not surprising because we were more prone to suggest a cryopreservation for lower sperm count but, interestingly, we did not find any significant difference for cryptozoospermic patients. The mean sperm count was also higher in the secured ICSI group compared to the non secured ICSI group for the whole panel of patients. It could be explained by the decreasing trend in the mean sperm count along the time of the study (Additional file 1: Figure S1) considering the fact that most of the secured ICSI were realized in the second part of the study.

### Are the frozen straws frequently used by the oligozoospermic patients?

Few of our patients used their frozen sperm on the day of ICSI, resulting in a 5.9% rate of use. Most of the studies available in the literature concerning the rate of frozen sperm use concern sperm banking before cancer therapies [14, 15, 18]. The rates observed vary from 2.7 to 27%. If we compare our rate of use to the one observed by Koscinski et al. among severe oligozoospermic patients, our results are in accordance (5.9% in our study vs 5.6% in their study). They observed a higher rate of use in case of cryptozoospermia when compared to very severe or severe oligozoosermia (our number of attempt is too small to allow a robust comparison of this point). The rate of thawing sperm for ICSI in case of oligozoospermia is even lower in the study by Ping et al. (3.0%, 2/66) [17] but no information is available in their study concerning the distribution of their patients according to the severity of the oligozoospermia. Moreover, since sperm banking is not costless in China, there is certainly a major bias of inclusion in their study The difference in rate of use between cancer situations and oligozoospermic situations may be explained by a higher risk of azoospermia in case of gonadotoxic treatment combined with a longer period of analysis in case of sperm banking before cancer treatment. We were not able to follow the evolution of this rate because of the shortness of our study and the low rate of use. This rate of use also highlights the need to better determine clinical and/or biological recommendations for sperm banking in case of severe oligozoospermia. We have defined our limit for such proposal, below 1Million/mL for the sperm count and within this limit, the rates of use are not higher in the cryptozoospermia group than in the mild oligozoospermia group. It should be useful to consider other parameters such as couple’s age, pathologies and sterilizing factors, and/or the hormonal status (FSH, LH, testosterone, inhibine B) of the men to decide if sperm banking has to be proposed and not focus only on the sperm count. A prospective study including all the previously mentioned parameters may be of relevance to address this point.

### Do the patients have the same chances of pregnancy even when sperm parameters are very low?

The results of ICSI for the whole cohort of patients are similar to those in the literature [7]. When we compare the results according to the sperm count, we find significant differences in the cleavage rate and the number of frozen embryos while no difference is observed in age, neither for women nor for men. It is not surprising that oligozoospermic patients have better outcomes than cryptozoospermic ones, this has already been described: Strassburger *et al*. [19] found significant decrease for the number of 24 h-4 cells embryos and the number of frozen embryos in case of very low sperm count. They also observed a significant decrease in the pregnancy rate, but only for cryptozoospermia group. We do not observe such difference for pregnancy and birth rate between the three groups. No significant difference in the ICSI outcome was found between frozen sperm and fresh ejaculated sperm. Although, the straws were used in a very few cases, others observed the same results with ejaculated or surgically retrieved spermatozoa [20–22].

### Repeated ejaculated sperm freezing or TESE?

When no spermatozoon is found the day of a non-secured ICSI, several choices appear: the patient can undergo a TESE (Testicular or Epididymal Sperm Extraction) if it has been planned on the same day and if TESE is not possible, the oocytes have to be vitrified [23, 24]. The question will then concern the possible use of these oocytes either through a planned TESE protocol or through a secured ICSI. The very low rate of use of the frozen ejaculated spermatozoa demonstrates a low risk of azoospermia on the day of ICSI in balance with high time consuming procedure of repeated sperm freezing. Moreover, the chances of pregnancy appear to be the same if ICSI is done with fresh or frozen sperm even when the spermatozoa come from the testis. We thus can wonder whether systematically planned TESE in case of cryptozoospermia or very severe oligozoospermia should be preferred to the repeated sperm freezing procedure. The fertilization or pregnancy rates of TESE-ICSI are similar to ICSI with ejaculated sperm [25–28] but TESE is at risk of testicular hematoma, hematocele, infection or further hypogonadism [29–32]. Moreover, the question about the risk for the descendants is still opened, because of the higher rate of aneuploidy [33, 34] or abnormal epigenetic content of these spermatozoa [35, 36]. Most of the studies report around 50% success of sperm retrieval [37–43]. TESE can be repeated to increase the chances to retrieve sperm [44] but the clinical risks mentioned above remain. Thus if possible ejaculated sperm freezing still remains simpler. To better evaluate the real risk of azoospermia in case of cryptozoospermia or severe oligozoospermia, multicenter prospective surveys should be done.

## Conclusion

Cryopreservation in case of severe oligozoospermia and cryptozoospermia is a practice more and more often offered to these patients. It allows avoiding the cancellation of the ICSI or the planning of a synchronous TESE; In our hands and considering the low frequency use of the thawed sperm, the chances of pregnancy and birth appear the same with fresh ejaculated or frozen-thawed sperm. The rate of use of the frozen sperm being quite low, we probably need to better characterize the patients who are really at risk of sperm decrease in order to avoid useless freezing. We also need to plan long term prospective studies to evaluate the real risk of azoospermia for these patients.

## Authors’ information

JB: Internship in Medical Biology in the Laboratory of ART, University Hospital of Grenoble, France.

SB: Biologist in the Laboratory of ART, University Hospital of Grenoble, France.

CTC, UB and SH: MD specialized in Medical Biology in the Laboratory of ART, University Hospital of Grenoble, France.

SB and SH: PhD, University Joseph Fournier in Grenoble, France; Team Andrology, Genetic and Cancer, AGIM-FRE 3405, Faculty of Medicine, Grenoble, France.

SS: Bachelor student in the Laboratory of ART, University Hospital of Grenoble.

PG: MD in the Department of Medical Information, University Hospital of Grenoble, France; PhD, UMR 5525, Technics of Medical Engineering and Complexity, Grenoble, France.

## Electronic supplementary material

Additional file 1: Figure S1.: Evolution of the mean sperm count and the distribution per group between 2004 and 2011. (DOCX 48 KB)
