# Supplementary figures and images for: In cryptozoospermia or severe oligozoospermia is sperm freezing useful?
Source: Basic Clin Androl. 2014 Oct 2;24:15. doi: 10.1186/2051-4190-24-15 (PMC4349690; doi:10.1186/2051-4190-24-15)

In cryptozoospermia or severe oligozoospermia, is sperm freezing useful?

*Additional file 1*


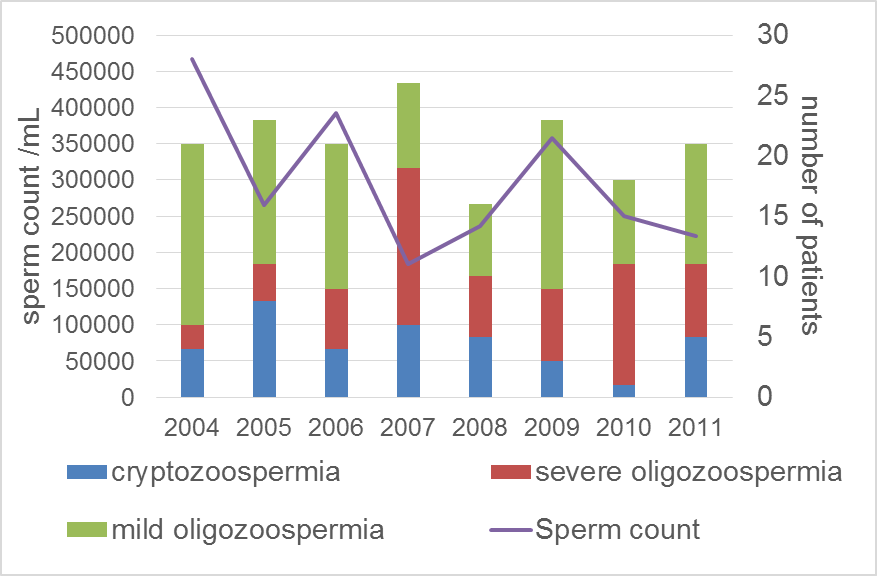

Supplement: Supplementary file 1 — Additional file 1: Figure S1.: Evolution of the mean sperm count and the distribution per group between 2004 and 2011. (DOCX 48 KB) [file 12610_2014_28_MOESM1_ESM.docx]
